# Antibiotics enhancing drug-induced liver injury assessed for causality using Roussel Uclaf Causality Assessment Method: Emerging role of gut microbiota dysbiosis

**DOI:** 10.3389/fmed.2022.972518

**Published:** 2022-09-09

**Authors:** Lihong Fu, Yihan Qian, Zhi Shang, Xuehua Sun, Xiaoni Kong, Yueqiu Gao

**Affiliations:** ^1^Central Laboratory, Department of Liver Diseases, ShuGuang Hospital, Affiliated to Shanghai University of Chinese Traditional Medicine, Shanghai, China; ^2^Institute of Infection Diseases, Shanghai University of Chinese Traditional Medicine, Shanghai, China

**Keywords:** gut microbiota, drug-induced liver injury, antibiotics, gut liver axis, probiotics

## Abstract

Drug-induced liver injury (DILI) is a disease that remains difficult to predict and prevent from a clinical perspective, as its occurrence is hard to fully explain by the traditional mechanisms. In recent years, the risk of the DILI for microbiota dysbiosis has been recognized as a multifactorial process. Amoxicillin-clavulanate is the most commonly implicated drug in DILI worldwide with high causality gradings based on the use of RUCAM in different populations. Antibiotics directly affect the structure and diversity of gut microbiota (GM) and changes in metabolites. The depletion of probiotics after antibiotics interference can reduce the efficacy of hepatoprotective agents, also manifesting as liver injury. Follow-up with liver function examination is essential during the administration of drugs that affect intestinal microorganisms and their metabolic activities, such as antibiotics, especially in patients on a high-fat diet. In the meantime, altering the GM to reconstruct the hepatotoxicity of drugs by exhausting harmful bacteria and supplementing with probiotics/prebiotics are potential therapeutic approaches. This review will provide an overview of the current evidence between gut microbiota and DILI events, and discuss the potential mechanisms of gut microbiota-mediated drug interactions. Finally, this review also provides insights into the “double-edged sword” effect of antibiotics treatment against DILI and the potential prevention and therapeutic strategies.

## Introduction

Drug-induced liver injury (DILI) is an underappreciated adverse drug reaction, in that the diagnosis of it still relies on the causality assessment, and that the Roussel Uclaf Causality Assessment Method (RUCAM) (1, 2) is the most commonly used scale recommended by various liver and Gastrointestinal associations (3–5). DILI can mimic features of various liver diseases, such as acute liver failure, drug-induced autoimmune hepatitis, and drug-associated fatty liver disease. The dramatic increase in drug-induced acute liver failure, setbacks in anti-tumor treatment, and herbal-related liver injury have raised global public health concerns (6). Specifically, the epidemiology and etiology of DILI differ in various countries and populations (7). The incidence rates range from 2.3 per 100,000 people in Sweden (8) to 19.1 per 100,000 people in Iceland (9). Even with rigorous preclinical toxicology tests, DILI events still occur unpredictably (5).

Since their invention in 1928, antibiotics have become life-saving medicines (10). Removal of bacterial taxa involved in the occurrence and progression of liver injury by vancomycin could alleviate liver disease in recent years (11, 12). However, the irrational use of antibiotics is a serious public health issue, and antibiotics appear to be a common cause of DILI, according to the etiology studies on causative agents tested for causality by RUCAM (13–15). Recently, antibiotics have been reported as the major agents responsible for DILI events in COVID-19 patients, which were the second only to antiviral drugs (16) (Supplementary Table 1). The drug-drug interactions (DDIs) mediated by the GM may trigger unpredictable adverse effects known as idiosyncratic DILI (iDIL) events (17). Notably, the use of antibiotics can also be involved in the protection and detoxification of DILI, and the mechanism and clinical manifestation of these two opposite situations may microbiologically share some common features as well as diversities (12, 18).

Metabolism has an overall effect on generating new toxicity or eliminating drug toxicity (19). Aside from the liver, the microbiota dispersed throughout the human body, especially in the intestine, also plays a crucial role in drug metabolism (20, 21). On the other hand, genomics links human genome variation to the unpredictability of DILI. Genome-wide association studies (GWAS) is a well-established field revealing how human leukocyte antigen (HLA) alleles or non-HLA variants increase susceptibility to DILI (22, 23). Nevertheless, their application in the diagnosis and management of DILI has been difficult (24). The microbial genome is considered to be the second human genome. The concept of pharmaceutical microbiomes brings a prospective approach to understand and address drug safety issues, as it is a modifiable pharmacogenomics (25).

Gut microbiota (GM) is an abundant and complex ecosystem containing 10^13^ microorganisms, mainly including Firmicutes (79.4%), Bacteroides (16.9%), Actinobacteria (2.5%), and Proteobacteria (1%) (26, 27). GM formation is influenced by multivariate determinants, such as host genetic factors, diet and lifestyle, environments, and oral drug use (28–30). Antibiotics appear to be a correlative drug for the microbiota remodeling (31), especially in newborns (32). GM and its metabolites are significant partakers in liver physiological functions, such as energy metabolism regulation, immune regulation, and modulation of resistance to infection (33, 34), which may also result in liver diseases such as inflammation, hepatic steatosis, and fibrosis (35–38).

In recent years, a model based on sterile or antibiotic-supplemented microbiota-depleted rodents has been used to explore the role of microbial intervention in experimental pharmacomicrobiomics. Summarizing the results of these experiments will help us to further understand the interaction between antibiotics and microorganisms, as well as their relationship with drug-induced hepatotoxicity. However, no past review had comprehensively examined whether GM could mitigate or aggravate DILI. This review will provide an overview of the current evidence between GM and DILI events, and discuss the potential mechanisms underlying the gut microbiota-mediated drug interactions. Finally, this review will also provide insights into the “double-edged sword” effect of antibiotics treatment against DILI and the potential prevention and therapeutic strategies. Finally, we hope that this frontier and conceptual research can at least provide a different tack to better understand the mechanism of iDILI, and find ways to prevent or treat it.

## The role of gut microbiota in drug-induced liver injury

### Gut-liver axis is the structural basis of drug-induced liver injury

Numerous viewpoints have emphasized the importance of a balanced intestinal microbiome in liver physiology and pathology (39). GM forms an axis with the liver primarily through a portal circulation, known as the gut-liver axis (40). This symbiotic relationship allows GM and its metabolites to be transferred from the gastrointestinal tract to the liver. In return, the liver secretes bile acids and antibodies to the intestinal lumen to regulate the composition and distribution of microbiota (41).

A functional gut-liver axis relies on a complete and solid intestinal barrier. So, there are several layers of defense that make up the intestinal barrier. The outermost layer is the mucus barrier, containing the bacterial colonization layer and the adhesive aseptic layer (42). In addition to immobilizing bacteria, the mucus barrier is also a source of nutrients for bacteria. For example, Akkermansia municiphila is a potential hepatoprotective bacterium that can maintain its growth by degrading mucins (43). The second layer is the epithelial barrier formed by a monolayer of epithelial cells, providing protection through its chemical and physical stability. Adjacent epithelial cells are closely connected by tight junctions, forming a physical barrier (44). Antimicrobial peptides and secreted IgA on the intestinal mucosa can block intestinal immune responses induced by microbial pathogens stimulation. Interactions among epithelial cells, immune cells, and mesenchymal cells create the intestinal mucosal ecological network that enables intestinal homeostasis (45). Gut vascular barrier (GVB) is another layer that prevents bacteria and their metabolites from entering the portal circulation when the outer mucus and epithelial barrier are broken (46).

Disruption of the three-layer barrier increases intestinal permeability, and the translocation of bacterial and microbial metabolites to the liver plays a key role in the pathogenesis of DILI (47, 48). Antigens derived from pathogenic microorganisms or drugs, such as lipopolysaccharides (LPS), cause microbiota-associated molecular pattern (MAMP) to activate nuclear factors κB (NF-κB) through toll-like receptors (TLRs) and nod-like receptors (NLRs), subsequently the released inflammatory cytokines and chemokines enter the portal circulation and reach the liver (49), where they trigger the proinflammatory cascade (50, 51). These antigens also lead to a series of excessive hepatic innate or acquired immune activation, such as NK cells, macrophages, and the release amounts of proinflammatory cytokines (52).

Interestingly, barrier damage may cause the involvement of activated stellate cells in fibrosis and, in turn, cirrhosis allows GM to enter the portal-venous circulation by destroying these barriers (42, 53). Cytokines downregulate the expression of tight junction proteins, thus changing the tight junction, of which the increase of intestinal permeability may be the mechanism (54).

## Composition and metabolites of gut microbiota and drug-induced liver injury

DILI is considered the consequence of a combination of variable host or non-host risk factors (55, 56), which may act by influencing the gut microbiota composition and metabolites (Table 1). Numerous studies have indicated that the reduced abundance of GM species and genes are the drivers of individual susceptibility to DILI (37, 52, 57–60). Microorganisms are enriched in genes encoding various enzymes which influence drug metabolism and increase the potential for liver injury (61, 62). Yip et al. (59) detected that Lactobacillus, Bacteroides, and Enterobacteriaceae, which would produce β-glucuronidase, were enriched in the strong responder groups (AST elevation ≥ 3 measurements) compared to the non-responder groups. Meanwhile, administration of β-glucuronidase to rats prior to administration of tacrine further confirmed it enhanced the potential hepatotoxicity induced by tacrine. In addition, host genes can also shape GM composition (35, 63). Previous studies have reported that modulation of key hepatic cytochrome enzymes expression results in individual differences in pharmacokinetics (64, 65). The expression of cyp3a11 was significantly higher in male mice than in female mice, whereas in mice lacking GM, the gender difference and cyp3a11 expression were approximately the same in both genders, indicating that gender differences and the composition/function of GM may importantly affect the patient’s response to drugs. Another microbiome-circadian rhythm study reported that circadian changes in GM could mediate different susceptibility to APAP-induced liver injury. Meanwhile, alcohol abuse would also aggravate the likelihood of DILI (4, 66). A recent review showed that alcoholic liver disease resulted in small intestinal bacterial overgrowth (SIBO), such as *E. coli*, and *Enterococcus* spp. (39). *E. coli*, *Enterococcus* spp. has been confirmed to generate 1-phenyl-1,2-propanedione (PPD), a microbial metabolite that could synergistically reinforce APAP-induced liver injury (37).

**TABLE 1:** List of main alteration of metabolites/composition in drug induced liver injury.

References	Drug	Alter of metabolites/composition
Clayton et al. (113)	Acetaminophen	β-glucuronidase/Firmicutes, Bacteroides
Gong et al. (37)	Acetaminophen	1-phenyl-1,2-propanedione/Escherichia coli, Citrobacter freundii
Yildirim et al. (114)	Neomycin, ampicillin, metronidazole	Enterobacteriales, anaerobic bacteria, Clostridiales phylum
Miao et al. (60)	Scutellarein	Enterococcus
Sun et al. (115)	Antithyroid	Prevotellaceae_UCG-003, Oscillibacter, Rikenellaceae_RC9
Zheng et al. (12)	Acetaminophen	P-cresol/Clostridium difficile
Yip et al. (59)	Tacrine	β-glucuronidase/Lactobacillus, Bacteroides, Enterobacteriaceae
Xia et al. (34)	Acetaminophen	SCFA/Oscillibacter, Colidextribacter, Mucispirillum
Yin et al. (116)	D-galactosamine	Proteobacteria, Blautia, Romboutsia, Parabacteroides, UCG-008, Parasutterella, Ruminococcus, norank_f: Lachnospiraceae, Eubacterium_xylanophilum, Oscillibacter, Eisenbergiella.

Apart from PPD, microbial metabolites such as bile acids, para–cresol, lysozyme, and lysophosphatide play pivotal roles in drug metabolism pathways in the liver (37, 57, 58, 67, 68). According to available studies, trigeminal, campesterol, and lithocholic acid varied significantly with the severity of liver injury (69). P-cresol produced by Clostridium difficile would increase toxicity by competing for the glutathione-dependent detoxification of phenolic drugs such as acetaminophen (68). In addition, abnormal metabolism of bile acids (BAs) would lead to decreased activation of nuclear receptors farnesoid X receptor (FXR) and TGR5 in ileal, thereby exacerbating hepatic steatosis and inflammation (70, 71).

## The role of antibiotics in gut microbiota

DILI is one of the adverse drug events (ADEs) following inappropriate or appropriate use of antibiotics (72). Nevertheless, emerging evidence has suggested that the administration of antibiotics had both negative and positive influences on the initiation and progression of DILI (Table 2). Positive influences include that hepatotoxic drugs no longer induced liver damage in mice after the mice were pretreated with antibiotics, while further studies found that it may be because the GM that causes liver inflammation was removed (37, 57). Here, we preliminarily focus on the mechanisms underlying the negative effects of antibiotics treatment on DILI.

**TABLE 2:** List of main studies in animals associating antibiotics treatment and drug induced liver injury.

References	Type-of animal used	Courses-of antibiotic treatment (day)	Antibiotic treatment	Combined treatment	Main findings in gut microbiota	Effect on drug-induced liver injury	Main mechanisms involved
Yip et al. (59)	Male C57BL/6 mice	7	Ampicillin, Neomycin, Metronidazole, Vancomycin	Triptolide	Depletion of intestinal flora	Aggravate liver injury	Dysregulation of arachidonic acid metabolism
Lama et al. (88)	Male C57BL/7 mice	15	Ceftriaxone	-	Diversity↓	Dysbiosis and bacterial translocation into the liver, triggering hepatic inflammation	The expression of Toll-like receptor 4 protein and Myeloid differentiation primary response
Yildirim et al. (114)	Male Sprague–Dawley rats, high-fat diet	14	Neomycin, Ampicillin, Metronidazole	Melatonin	Enterobacteriales↑, Anaerobic bacteria↑, Clostridiales phylum↓	Enhanced hepatic injury and dysfunction	Increased neutrophil accumulation to liver
Zheng et al. (12)	Male SD and Wistar rats and C57 mice	4	Vancomycin	Acetaminophen	The activity of β-glucuronidase↓, Firmicutes↓, Bacteroides↓	Attenuation on AP-induced liver injury	Decreased hepatic Cyp7a1 expression. Increased GSH level. Up-regulated mRNA expression of Nqo-1 and Gclc gene, and downregulated Tnf-a and Il-1b
Kolodziejczyk et al. (18)	Male C57BL/8 mice	14	Ampicillin, Neomycin, Metronidazole, Vancomycin	Acetaminophen and Thioacetamide	Depletion of Intestinal flora	APAP and TAA-induced liver toxicity were attenuated	Suppress MYC-dependent program
Li et al. (107)	Male C57BL/6 mice, ethanol-fed	3	Terramycin, Erythromycin	Berberine	Depletion of Intestinal flora	Berberine did not show any positive effect on alcohol-induced hepatic injury	Inhibited the activation of granulocytic-myeloid-derived suppressor cell-like population
Miao et al. (60)	Male BALB/c mice	35	Ampicillin, Neomycin sulfate, Metronidazole, Vancomycin	Carbon Tetrachloride, Scutellarein	Bifidobacterium↑, Lactobacillus↑, Enterococcus↓	Reversed the hepatoprotective effect of Scutellarein in Carbon Tetrachloride-induced chronic liver injury	Activated CYP2E1 expression and worsened CYP2E1-mediated lipid peroxidation and oxidative stress
Luo et al. (52)	Male BALB/c mice	8	Ceftriaxone	-	Damages of gut microbial barrier	It mediates the occurrence of chronic hepatitis	Activation of immunocytes, such as NK cells, γδT cells, NKT cells et al.
Blake et al. (117)	Male C57BL/7 mice	7	Neomycin, Ampicillin	Anti-CD40 and anti-CD137 immunotherapies	Depletion of Intestinal flora	Significantly reduced the liver damage after immune agonist antibodies treatment	Modulates anti-CD40-induced changes to lipid and bile acid metabolism in the liver
Gong et al. (37)	Male C57BL/6 mice	3	Vancomycin, Neomycin sulfate, Metronidazole, Ampicillin	Cisplatin	Depletion of Intestinal flora	Cisplatin hepatotoxicity was prevented	The phosphorylation of proteins involved in the JNK and p38 pathways
Huang et al. (58)	Male Lister hooded rats	3	Vancomycin, Imipenem	Tacrine	β-glucuronidase–producing bacteria such as Bacteroides and Enterobacteriaceae ↓	The susceptibility of Tacrine induced hepatotoxicity was significantly reduced	Ischemia or reperfusion
Luo et al. (89)	Male C57BL/6 mice, high-fat diet	7	Penicillin	Cassiae Semen extract	Diversity↓	TC↑, FFA↑, ALT/AST↑	Hepatoprotective efficacy of CS was inhibited or eliminated
			Metronidazole			TC↑, FFA↑, TG↑	
			Clindamycin			TC↑, FFA↑, ALT↑	
			Vancomycin			TC↑, FFA↑, LDL-c↑, ALT/AST↑	
			Neomycin			TC↑, FFA↑, ALT/AST↑	
			Penicillin, Metronidazole, Clindamycin, Vancomycin, Neomycin			TC↑, FFA↑, LDL-c↑, ALT↑	

**FIGURE 1:**
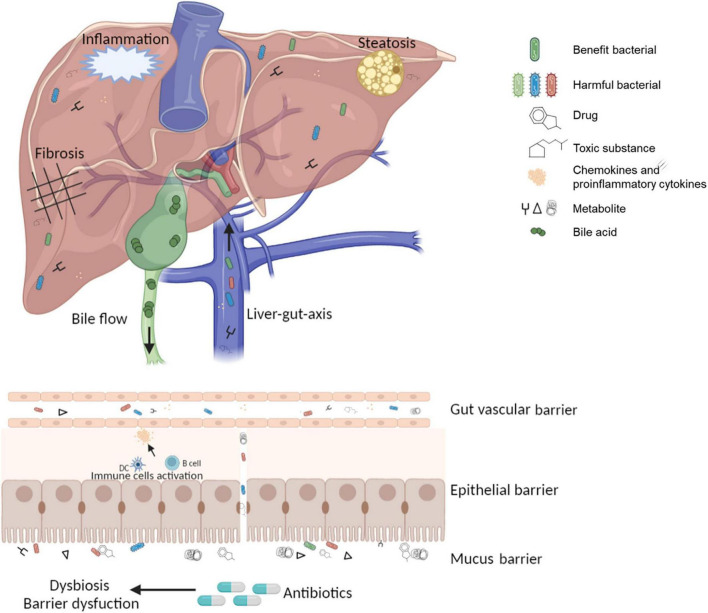
The pathological state of the gut-liver-immune axis and gut microbiota under antibiotic treatment.

Since the discovery in 1950 that terramycin can affect the GM in human (73), there has been increasing evidence that antibiotics may cause dysbiosis through the reactive proliferation of potentially pathogenic microorganisms, depletion of beneficial bacteria, loss of α-diversity, and leakage of gut activities (73). Dysbiosis causes irreversible variation and functional impairment of GM at the gene or protein level, leading to disturbances of the immune system in intestinal epithelial cells, ultimately affecting hepatic metabolism (74) (Figure 1). However, it must be emphasized that some antibiotics had effects on intestinal barrier function and resulted in major changes in the microbiome, but not all, for example, metronidazole treatment had no effect on the microbiota (75). Due to different classes of antibiotics and individual responses, these effects could leave a few days or permanent imprints in the intestinal environment (76, 77), which might explain the significant difference in the onset time and course of various antibiotic-mediated liver injury.

Several human cohort studies have demonstrated the association between antibiotics use and changes in microbial composition and function (29, 78). Antibiotic therapy aims to eliminate pathogenic bacteria, however, this microbial clearance will also reduce the beneficial commensal bacteria, which has important pathological implications for the liver (36, 79). β-lactam antibiotics and the antibiotics cocktails were observed to increase the Bacteroidetes/Firmicutes ratio and decrease the microbial diversity. In particular, the abundance of Firmicutes and Actinobacteria (31, 60, 80), which contained bacteria that played an important role in reducing oxidative stress, inflammation, and liver-protective properties, such as Bifidobacterium and Lactobacillus (81–83). Noteworthy, antibiotics also increase the abundance of pathogenic bacteria, such as Enterococcus, which may exacerbate the progression of liver diseases (84).

Moreover, a handful of studies have proposed that flora changes could also alter the distribution of bacterial metabolites (85). For example, ciprofloxacin, a novel quinolone antibiotic with antimicrobial activity against lithocholic acid-producing bacteria, might lead to a reduction of lithocholic acid in the liver, thereby reducing the expression of the hepatic drug-metabolizing enzyme CYP3A (86).

Antibiotics can lead to impairment of the intestinal biological barrier and affect intestinal permeability. Ceftriaxone and ciprofloxacin have been proven to cause severe damage and histomorphological changes in the intestinal villus wall in an animal model (87). Expression of Toll-like receptor 4 protein and Myeloid differentiation primary response (Myd) 88 mRNA, which activated the NF-κB signaling pathway, was increased in both gut and liver after ceftriaxone treatment (88). In a separate study (89), lower expression of occludin and occludens-1 (ZO-1) mRNA in the ileum, which was the most critical component in tight junction proteins and functional organization to protect intestinal barrier permeability and epithelial integrity, was seen in different groups treated with ampicillin, vancomycin, neomycin, metronidazole, and mixtures of them (90–92).

On the other hand, the leakage of the gut activates the overexpression of nitric oxide (NO) synthesis (39, 93). NO induce the enhancement of tubulin nitration and oxidation, leading to further disruption of the barrier function by the microtubule cytoskeleton. In addition, increased NO synthesis leads to oxidative stress in hepatocytes (94). Numerous studies have demonstrated that lipopolysaccharide (LPS) spilled into the systemic circulation through the permeable intestine, resulting in hepatic immune activation (95).

Another related concept is colonization resistance, which plays an important role in preventing pathogen colonization and protecting intestinal function (96). Some animal and human studies have suggested that antibiotics could sabotage this ability (97, 98). However, how this destruction affects the occurrence of liver diseases is not completely understood.

## Administration of antibiotics as a factor in drug-induced liver injury

### Sole antibiotic treatment

A couple of studies have highlighted the association between antibiotics and DILI by affecting GM. Luo et al. (52) have documented that ceftriaxone significantly reduced GM diversity, increased the levels of pathogenic bacteria such as Firmicutes, Tendericutes, and Vibrio bacteria, caused damage to intestinal barrier, promoted the expression of LPS, and activated liver lymphocytes. The H&E staining of the liver showed hepatic steatosis and hepatitis. And the expression levels of ALT, AST, IL-6, and TNF- α in serum increased. These findings were consistent with similar findings in other studies of different classes of antibiotics (88, 99). In another study (80), it was confirmed that various antibiotic combinations had different effects on host BA metabolism. In particular, a stronger effect was observed in combination of two antibiotics than in single antibiotics.

### Antibiotics combined with chemical agents

To date, there are not enough studies showing that the combination of antibiotics and chemical agents might lead to potential DDIs and impact the development of DILI (100, 101). Yoo et al. (102) indicated that antibiotic treatment of patients taking lovastatin might lead to adverse pharmacokinetic effects by suppressing GM. The failure of plasma cholesterol control exerted an influence on hepatic steatosis (103). Besides, hepatotoxic chemicals could also be exposed to the environment, and in an animal model, an antibiotics cocktail containing ampicillin, vancomycin, neomycin, and metronidazole increased polychlorinated biphenyls-induced inflammation but decreased hepatic fibrosis (91).

### Antibiotics combined with herbal agents

Similarly, the disturbance of GM by antibiotics also modulated the susceptibility to natural compounds-induced transaminitis (33, 104). Clearance of GM before Triptolide treatment could increase bile acids and long-chain fatty acids in plasma and liver (58). The accumulation of bile acids may lead to necrosis and apoptosis of hepatocytes, and stimulate the release of TNF-α, IL-6 and IL-8 (105, 106). The study also found that the mRNA levels of inflammatory indicators in the liver were significantly elevated in the TP + antibiotic group, but downregulated in the antibiotics alone group, which has identified the risk of TP and its preparations administrated in combination with antibiotics. However, co-administration with propionate almost eliminated this inflammatory response.

Another hypothesis was that antibiotics interfered with GM and significantly reduced or even reversed the hepatoprotective effects of other drugs. Berberine did not show any positive effect on alcohol-induced liver injury in an antibiotic molded pseudo-germ-free (PGF) mouse model (107). Scutellarein was a herbal flavonoid thought to have hepatoprotective potential (108), however, Miao et al. (60) confirmed that when scutellarein was used in combination with antibiotics, it activated IκBα/NF-κB pathway, CYP2E1 expression, and aggravated CYP2E1-mediated lipid peroxidation and oxidative stress through intestinal ecosystem disorder (60), which was consistent with another study (89). The hepatoprotective effects of Cassiae Semen on mice were weakened or eliminated in different classes of antibiotics groups. The antibiotic-induced liver injury needs to be vigilant, especially during hepatoprotective therapy.

## Recommendation on the safety of combination therapy: Perspective of gut microbiota

RUCAM, as a well-established diagnostic scale, can accurately assess the case of iDILI through the well-described clinical characteristics. However, the variability of clinical characteristics of iDILI, the difficulty in performing rigorous mechanistic studies in humans, and the lack of an animal model of experimental iDILI that can mimic the genetic requirements of human iDILI, make it impossible to obtain satisfactory and specific biomarkers for individuals with iDILI. The changes of different antibiotics on different GM provide a new idea and method for the experimental animal model, which can imitate the genetic requirements of iDILI patients. Collecting biological samples such as feces from iDILI patients for omics analysis is the most common and meaningful means to identify biomarkers, but the screening of the iDILI cases must be careful. Further investigations in patients with idiosyncratic DILI with high causality gradings based on the use of RUCAM is an integral evaluating mechanistic step (109).

Antibiotics aggravate the susceptibility to DILI by causing dysbiosis and barrier dysfunction affecting the disposal and action of other drugs (25). For liver injury induced by drug combination, DDIs should be explored, and the detailed mechanism could help prevent unexpected accidents and determine appropriate diagnosis and treatment. The drug combination therapy might cause DDIs through the regulation of drug metabolic enzymes and drug efflux pumps. However, when drugs were used in combination with antibiotics, subsequent changes in xenobiotics metabolism mediated by gut microbial enzymes would occur (110). Similarly, even if primary antibiotic treatment was safe, increased therapy might also cause liver inflammation (59), which suggested that follow-up of liver function test was essential during the administration of drugs such as antibiotics that affect intestinal microorganisms and their metabolic activities. In addition, we recommended that avoiding the risk for liver injury should be considered when establishing individual therapy, including the nature, duration and intervention time of antibiotics, which would affect the efficacy and toxicity of drugs.

Although some drugs have surprising curative effects, they also have the risk of inducing the outbreak of liver failure (57, 111). Altering the GM to reconstruct the hepatotoxicity of drugs by exhausting harmful bacteria, and supplementing probiotics/prebiotics or fecal microbiota transplantation are therapeutically potential. Monitoring the composition and metabolic activity of GM can provide a new target for early diagnosis or prevention and treatment strategies for DILI (112). Antibiotics are the most significant microbiome-targeted drugs to alleviate drug-induced liver failure (18). Pharmacological mechanisms include reducing bacterial density, eliminating target harmful bacteria, inhibiting secondary bacterial proliferation, and reducing bacterial translocation. But the appropriate target remains an unknown problem, which can be accomplished in multiomic integration, including combining transcriptomics, genomics, and metabolomics. Disappointingly, most studies used antibiotic mixtures or broad-spectrum antibiotics to deplete all GM, which could only explain the causal relationship between GM and DILI. Since the GM have been proven to have a protective effect on a variety of diseases, whether it could target specific flora had little practical significance for the prevention and treatment of DILI. Therefore, the therapeutic significance of “sterilization” to human health needs to be further explored. And the choice of antibiotics is also crucial. The effects of antibiotics on the overall composition of the microbiome and the downstream effects on the microbiome and host should be clarified in research. Another potential therapeutic approach is probiotics/prebiotics, for instance, Myxophilic bacteria has potential therapeutic value to reduce oxidative stress and inflammation in the liver by modulating GM composition and metabolic function, thereby alleviating APAP-induced liver injury (34).

## Conclusion

In general, the heterogeneous response of drugs presents significant challenges for drug development and patient management. With the intention to establish a valid diagnosis, the use of a diagnostic scale such as RUCAM is recommended. Orally administered drugs may become toxic after being metabolized by GM before entering the liver. Combination with antibiotics leads to DDIs mediated by intestinal metabolism, suggesting that the possibility of hepatotoxicity caused by this combination should be vigilant. Other drugs metabolized by intestinal microbial enzymes may also produce similar antibiotic-induced pharmacokinetic effects. Therefore, a wider range of drugs need to be further studied (102). In the foreseeable future, the regulation of GM to improve treatment will ameliorate clinical practice.

## Author contributions

LHF and YQG contributed to the concept and design of the study. LHF and YHQ wrote, edited, and reviewed of the manuscript. XNK and XHS revised the manuscript. ZS designed the tables and figures. All authors contributed to manuscript revision, read, and approved the submitted version.
