# Supplementary material for: Antibiotics enhancing drug-induced liver injury assessed for causality using Roussel Uclaf Causality Assessment Method: Emerging role of gut microbiota dysbiosis
Source: Front Med (Lausanne). 2022 Sep 9;9:972518. doi: 10.3389/fmed.2022.972518 (PMC9500153; doi:10.3389/fmed.2022.972518)
Supplement: Supplementary file 1 [file Table_1.DOCX]

Supplementary Material

# Supplementary Figures and Tables

## Supplementary Table

**Supplementary Table 1**. List of antibiotics in reports that applied RUCAM in suspected DILI cases.

| **Ref.** | **Cases**  **(n)** | **Type of antibiotics** | **Population** | **RUCAM based scores** |
| --- | --- | --- | --- | --- |
| (1) | 55 | Tigecycline | Inpatients | ≥6 |
| (2) | 938 | Various antibiotics | Paediatric patients | Unmentioned |
| (3) | 78 | Amoxicillin/Clavulanate | Registry cases | ≥3 |
| (4) | 2 | Amoxicillin/Clavulanate | Inpatients | ≥6 |
| (5) | 80 | Various antibiotics | Inpatients | 5–8 |
| (6) | 43 | Various antibiotics | Inpatients | Unmentioned |
| (7) | 51 | Flucloxacillin | Inpatients | ≥6 |
| (8) | 2 | Amoxicillin,  Amoxicillin/Clavulanate | Inpatients | 9 |
| (9) | 1 | Cephalexin | Inpatients | 6 |
| (10) | 1 | Ciprofloxacin | Inpatients | 8 |
| (11) | 197 | Flucloxacillin | Inpatients | 11 |
| (12) | 9 | Daptomycin | Inpatients | Unmentioned |
| (13) | 1 | Azithromycin, Ceftriaxone | Inpatients | Unmentioned |
| (14) | 91 | Azithromycin, Ceftriaxone | COVID-19 patients | ≥4 |

## Reference

1. Shi X, Zuo C, Yu L, Lao D, Li X, Xu Q, et al. Real-World Data of Tigecycline-Associated Drug-Induced Liver Injury among Patients in China: A 3-Year Retrospective Study as Assessed by the Updated Rucam. *Front Pharmacol* (2021) 12:761167. Epub 2021/11/20. doi: 10.3389/fphar.2021.761167.

2. Ferrajolo C, Verhamme KMC, Trifirò G, ‘t Jong GW, Picelli G, Giaquinto C, et al. Antibiotic-Induced Liver Injury in Paediatric Outpatients: A Case-Control Study in Primary Care Databases. *Drug Safety* (2017) 40(4):305-15. doi: 10.1007/s40264-016-0493-y.

3. Lucena MI, Molokhia M, Shen Y, Urban TJ, Aithal GP, Andrade RJ, et al. Susceptibility to Amoxicillin-Clavulanate-Induced Liver Injury Is Influenced by Multiple Hla Class I and Ii Alleles. *Gastroenterology* (2011) 141(1):338-47. doi: <https://doi.org/10.1053/j.gastro.2011.04.001>.

4. Visentin M, Lenggenhager D, Gai Z, Kullak-Ublick GA. Drug-Induced Bile Duct Injury. *Biochimica et Biophysica Acta (BBA) - Molecular Basis of Disease* (2018) 1864(4, Part B):1498-506. doi: <https://doi.org/10.1016/j.bbadis.2017.08.033>.

5. Treeprasertsuk S, Huntrakul J, Ridtitid W, Kullavanijaya P, BjÖRnsson ES. The Predictors of Complications in Patients with Drug-Induced Liver Injury Caused by Antimicrobial Agents. *Aliment Pharmacol Ther* (2010) 31(11):1200-7. doi: <https://doi.org/10.1111/j.1365-2036.2010.04292.x>.

6. Hussaini SH, O'Brien CS, Despott EJ, Dalton HR. Antibiotic Therapy: A Major Cause of Drug-Induced Jaundice in Southwest England. *European journal of gastroenterology & hepatology* (2007) 19(1):15-20. Epub 2007/01/09. doi: 10.1097/01.meg.0000250581.77865.68.

7. Daly AK, Donaldson PT, Bhatnagar P, Shen Y, Pe'er I, Floratos A, et al. Hla-B*5701 Genotype Is a Major Determinant of Drug-Induced Liver Injury Due to Flucloxacillin. *Nat Genet* (2009) 41(7):816-9. doi: 10.1038/ng.379.

8. Fontana RJ, Shakil AO, Greenson JK, Boyd I, Lee WM. Acute Liver Failure Due to Amoxicillin and Amoxicillin/Clavulanate. *Digestive Diseases and Sciences* (2005) 50(10):1785-90. doi: 10.1007/s10620-005-2938-5.

9. Singla A, Hammad HT, Hammoud GM. Uncommon Cause of Acute Drug-Induced Liver Injury Following Mammoplasty. *Gastroenterology research* (2010) 3(4):171-2. Epub 2010/08/01. doi: 10.4021/gr2010.06.210w.

10. Unger C, Al-Jashaami LS. Ciprofloxacin Exposure Leading to Fatal Hepatotoxicity: An Unusual Correlation. *The American journal of case reports* (2016) 17:676-81. Epub 2016/09/23. doi: 10.12659/ajcr.899080.

11. Nicoletti P, Aithal GP, Chamberlain TC, Coulthard S, Alshabeeb M, Grove JI, et al. Drug-Induced Liver Injury Due to Flucloxacillin: Relevance of Multiple Human Leukocyte Antigen Alleles. *Clin Pharmacol Ther* (2019) 106(1):245-53. doi: <https://doi.org/10.1002/cpt.1375>.

12. Bohm N, Makowski C, Machado M, Davie A, Seabrook N, Wheless L, et al. Case Report and Cohort Analysis of Drug-Induced Liver Injury Associated with Daptomycin. *Antimicrob Agents Chemother* (2014) 58(8):4902-3. doi: 10.1128/AAC.03157-14.

13. Muhović D, Bojović J, Bulatović A, Vukčević B, Ratković M, Lazović R, et al. First Case of Drug-Induced Liver Injury Associated with the Use of Tocilizumab in a Patient with Covid-19. *Liver Int* (2020) 40(8):1901-5. doi: <https://doi.org/10.1111/liv.14516>.

14. Delgado A, Stewart S, Urroz M, Rodríguez A, Borobia AM, Akatbach-Bousaid I, et al. Characterisation of Drug-Induced Liver Injury in Patients with Covid-19 Detected by a Proactive Pharmacovigilance Program from Laboratory Signals. *Journal of Clinical Medicine* (2021) 10(19). doi: 10.3390/jcm10194432.
